# Temporary Shutdown of ERK1/2 Phosphorylation Is Associated With Activation of Adaptive Immune Cell Responses and Disease Progression During *Leishmania amazonensis* Infection in BALB/c Mice

**DOI:** 10.3389/fimmu.2022.762080

**Published:** 2022-01-25

**Authors:** Leandro G. Oliveira, Míriam C. Souza-Testasicca, Tiago Nery Queiroga Ricotta, Juliana P. Vago, Liliane M. dos Santos, Frederico Crepaldi, Kátia M. Lima, Celso Queiroz-Junior, Lirlândia P. Sousa, Ana Paula Fernandes

**Affiliations:** ^1^ Departamento de Análises Clínicas e Toxicológicas, Faculdade de Farmácia, Universidade Federal de Minas Gerais, Belo Horizonte, Brazil; ^2^ Departamento de Biologia, Instituto Federal de Minas Gerais (IFMG), Ouro Preto, Brazil; ^3^ Departamento de Morfologia, Instituto de Ciências Biológicas, Universidade Federal de Minas Gerais, Belo Horizonte, Brazil

**Keywords:** *L. amazonensis*, kinetics of ERK1/2 activation, inflammation, leishmaniasis infection, *L. braziliensis*, vaccine, treatment

## Abstract

*Leishmania* spp. infection outcomes are dependent on both host and parasite factors. Manipulation of host signaling pathways involved in the generation of immune responses is thought to be one of the most common mechanisms used by parasites for persistence within the host. Considering the diversity of pathologies caused by different *Leishmania* spp., it is plausible that significant differences may exist in the mechanisms of host cell manipulation by each parasite species, which may have implications when developing new vaccine or treatment strategies. Here we show that in *L. braziliensis*-infection in BALB/c mice, a model of resistance, activation of ERK1/2 coincides with the peak of inflammatory responses and resolution of tissue parasitism. In contrast, in the susceptibility model of *L. amazonensis*-infection, an early silent phase of infection is observed, detected solely by quantification of parasite loads. At this early stage, only basal levels of P-ERK1/2 are observed. Later, after a brief shutdown of ERK1/2 phosphorylation, disease progression is observed and is associated with increased inflammation, lesion size and tissue parasitism. Moreover, the short-term down-regulation of ERK1/2 activation affected significantly downstream inflammatory pathways and adaptive T cell responses. Administration of U0126, a MEK/ERK inhibitor, confirmed this phenomenon, since bigger lesions and higher parasite loads were seen in infected mice that received U0126. To investigate how kinetics of ERK1/2 activation could affect the disease progression, U0126 was administered to *L. amazonensis*-infected animals earlier than the P-ERK1/2 switch off time-point. This intervention resulted in anticipation of the same effects on inflammatory responses and susceptibility phenotype seen in the natural course of infection. Additionally, *in vitro* inhibition of ERK1/2 affected the phagocytosis of *L. amazonensis* by BMDMs. Collectively, our findings reveal distinct temporal patterns of activation of inflammatory responses in *L. braziliensis* and *L. amazonensis* in the same animal background and a pivotal role for a brief and specific shutdown of ERK1/2 activation at late stages of *L. amazonensis* infection. Since activation of inflammatory responses is a crucial aspect for the control of infectious processes, these findings may be important for the search of new and specific strategies of vaccines and treatment for tegumentary leishmaniasis.

## Introduction

Parasites of the genus *Leishmania* are dimorphic protozoa that are transmitted to humans or other mammalian hosts by the bite of a phlebotominae sand fly (1). *Leishmania* infection may evolve to a broad range of clinical manifestations and outcomes, varying from localized and self-healing cutaneous leishmaniasis (CL) to visceral leishmaniasis (VL), a fatal disease if not treated. Approximately 30% out of the 1.2 million new cases of CL reported each year worldwide occur in the Americas, and Brazil is among the 10 countries with the highest incidence rates of this clinical form (1). American tegumentary leishmaniasis (ATL) comprises some of the main clinical manifestations associated with infections by different species of *Leishmania*. *Leishmania braziliensis* is one of the most prevalent etiologic agents of ATL and may lead to CL, which is characterized by skin ulcers, usually localized at the site of the sand fly bite and mucosal leishmaniasis (ML), a severe form of TL which affects mucosa of the nose, throat and mouth. In localized cutaneous leishmaniasis (LCL) and ML, intense inflammatory responses and scant parasites coexist in lesions that may become chronic (2–4). Parasites of the *Leishmania mexicana* complex, including *Leishmania amazonensis* may also cause these ATL forms, in addition to diffuse cutaneous leishmaniasis (DCL), which is associated with disseminated non-ulcerative lesions, anergic immune responses and huge numbers of parasites in lesions (2).

The infection outcome in leishmaniasis is largely dependent on the interplay of several factors such as parasite species, vector biology, host genetics and immune system. In mouse models of *L. braziliensis* infection, including BALB/c, host immune response is characterized by early production of inflammatory mediators, complete activation of phagocytic and T cells that contribute to rapid and efficient parasite clearance leading to development of lesions that are small and mostly self-healing (2, 5). In contrast, most inbred strains of mice are commonly susceptible to *L. amazonensis* infection, even though the course of infection in each mice strain also presents striking differences. *L. amazonensis* infected BALB/c mice, for example, develop non-healing lesions that progress continuously for weeks, present an intense inflammatory infiltrate and high tissue parasitism (6). Although most *Leishmania* species can evade the immune system, *L. amazonensis* parasites are thought to be highly skilled in repressing host cell activation, to escape anti-microbial functions (7, 8). Indeed, early events triggering or suppressing inflammatory responses may be the key elements used by the widely diverse *Leishmania* species to escape from host defense mechanisms and survive, while leading to these wide clinical distinct diseases and immunopathology patterns.

Despite the considerable incidence and associated morbidity, therapeutic alternatives for leishmaniasis are either scant, toxic, and usually not effective, or still expensive for most patients (2). A prophylactic vaccine for human leishmaniasis is not currently available but seems to be the way to successfully control the disease since immunity generated by the infection in both mice and humans was shown to be long-lasting and able to protect against subsequent infections in the same species or different species of *Leishmania* (9, 10). A vaccine against leishmaniasis should be able to induce a potent Th1-type immune response capable of anticipating and outperforming the immune responses generated by the primary infection, therefore controlling parasite growth and the onset of the inflammatory responses that leads to pathology. Full understanding of the molecular pathways involved in the generation of immune responses during infection could determine the success of development of new vaccine and treatment alternatives, circumventing the mechanisms that *Leishmania* species display to escape host immune responses.

Activation of mitogen activated protein kinases (MAPKs) are listed as one of the most significant pathways associated with inflammatory responses, which promotes activation of T lymphocytes, production of cytokines and chemokines and the recruitment and survival of leukocytes. Among them, the extracellular regulated protein kinase (ERK) 1/2 pathway has been linked to growth, proliferation, and survival cell processes, which are essential for successful establishment of infection by several pathogens (11, 12). *Leishmania* species have been shown to influence key steps in the regulation of important molecules of cell signaling cascades, including activation of ERK1/2 and the consequent modulation of macrophage function *in vitro* (13–16).

It has been previously reported that lesion recovered amastigotes of *L. amazonensis* can induce ERK1/2 activation in bone marrow-derived macrophages (BMDMs) from BALB/c mice (17). It has also been proposed that amastigotes of *L. mexicana* complex can cause cysteine peptidase-dependent degradation of ERK and JNK, but not p38 (18). However, kinetics of ERK expression and phosphorylation has not yet been fully and comparatively addressed during *Leishmania* infections. Here, we compared the inflammatory responses at different time-points after infection of BALB/c mice by *L. braziliensis* or *L. amazonensis*, defined as resistance and susceptibility models of infection, respectively, and their association with the MEK/ERK signaling pathway.

## Materials and Methods

### Mice

Female BALB/c mice at five weeks of age were purchased from the mouse breeding facility of the Universidade Federal de Minas Gerais (Brazil). This study was carried out in strict accordance with institutional ethical guidelines (CEUA protocol number 240/2016).

### Parasites

*L. amazonensis* (IFLA/BR/67/PH8) and *L. braziliensis* (MHOM/BR/75/M2903 strain) were cultured in Grace’s insect medium (Sigma-Aldrich, USA) (pH 6.5) supplemented with 10% heat-inactivated FCS (Gibco, USA), 2 mM L-glutamine (Sigma-Aldrich), 100 U/ml penicillin, and 100 mg/ml streptomycin (Sigma-Aldrich) at 25 ˚C. Parasites were sub-cultured every week at 1 x 10^5^ parasites/ml. Periodically recovery from infected animals were performed and all the experiments were conducted with parasites with less than 15 passages in culture (19).

### *In Vivo Leishmania* Infection and U0126 Administration

BALB/c mice (3-5 mice per group) were inoculated in the left hind footpad with 1x10^5^ early stationary phase promastigotes of *L. amazonensis* or 1x10^7^ early stationary phase promastigotes of *L. braziliensis*, and lesion development was followed weekly with a digital micrometer (Western, Etilux, Brazil). The results were expressed as the difference between measures of infected and noninfected footpads. Mice received an intraperitoneal (i.p) administration of 3 mg/Kg/i.p. daily for seven consecutive days of the MEK/ERK inhibitor U0126 (1,4-diamino-2,3-dicyano-1,4-bis[2-aminophenylthio] butadiene - Cell Signaling Technology, Beverly, MA, USA). The U0126 solution was prepared in DMSO according to the manufacturer’s protocol and mixed with sterile saline before use. The control group of infected mice received the vehicle (DMSO) in equal proportion.

### *In Vitro L. amazonensis* Infection of Bone Marrow-Derived Macrophages and Inhibitor Administration

Bone marrow-derived macrophages (BMDMs) from BALB/c mice were obtained by differentiating bone marrow cells in complete DMEM supplemented with 10% of FCS and 20% of LCCM supernatant as previously described (20). After 7 days, BMDMs were collected and plated at 1x10^5^ cells/well onto a 16-well chamber slide. After 2 hours of incubation at 37°C in 5% CO_2_ non-adherent cells were removed. After 2h pre-incubation with U0126 (15 µM) or selumetinib (15 µM - Selleck Chemicals, Houston, TX), inhibitors were removed by washing with PBS and stationary phase promastigotes of *L. amazonensis* were added to the culture at a multiplicity of infection (MOI) of 5:1 parasite/cell ratio. After 3h of infection, cells were washed with PBS to remove extracellular parasites and incubated with fresh medium at 37°C in 5% CO_2_. Macrophage infections were evaluated at 3, 6, 24 and 48 hours after infection using Giemsa staining kit (Laborclin, Brazil), according to the manufacturer’s instructions. The number of infected and uninfected cells and the number of parasites present in infected cells were determined. A minimum of 200 macrophages per coverslip were examined.

### Antigen Preparation

Early stationary phase promastigotes from *L. amazonensis* or *L. braziliensis* cultures were pelleted by centrifugation and washed twice in PBS. Washed parasites were then resuspended in PBS and disrupted by sonication using 5 cycles of 1 minute. The preparations (AgLa or AgLb) were observed under a microscope for the presence of intact parasites and the protein content was determined by Bradford method (21).

### Lysate Preparation and Western Blot Analysis

The western blot analysis was performed as described elsewhere (22). The antibodies used were anti–β-actin (Sigma-Aldrich), anti-P-ERK1/2, anti-P-NF-κBp65/RelA and anti-cleaved Caspase-3 (all from Cell Signaling Technology). Densitometry analyses were performed using ImageJ software (National Institutes of Health, Bethesda, MD). Data were expressed in arbitrary units (AU) after normalization to the of β-actin values.

### Cytokine Profile Determination

At different periods of time post-infection animals were euthanized and the draining popliteal lymph nodes were collected. Lymph nodes were processed in a glass homogenizer and the obtained cell suspension had its concentration adjusted to 5 x 10^6^ cells/ml in DMEM supplemented with 10% of FCS. The cells were incubated in the absence or presence of stimulus (AgLa or AgLb: 50 µg/ml) for 48h at 37°C, 5%. The concentrations of IFN-γ and IL-10 in the cell culture supernatants were performed by ELISA using the specifics OptEIA™ Mouse Kit (BD Biosciences, USA) according to the instructions of the manufacturers.

### Estimation of Parasite Load

For parasite load determination, at different periods of time post-infection animals were euthanized and the infected paws were collected for parasite load determination. The limiting-dilution technique was performed as previously described (23). Briefly, the infected paws were mechanically homogenized in Grace’s insect medium supplemented with 10% heat-inactivated FCS, 2 mM L-glutamine, 200U/mL penicillin and 100 μg/ml streptomycin. Each homogenized sample tissue was serially diluted (1/10) in a 96-well flat-bottomed microtiter plate containing the same medium (in duplicates). The number of viable parasites was determined from the highest dilution at which promastigotes could be grown up to 7 days of incubation at 23°C.

### Histological Analysis

The skin samples of the plantar surface of mouse paws were collected and fixed in 10% neutral buffered formalin (pH 7.2). Sampling processes and the determination of inflammatory infiltrate scores were performed as previously described (22).

### Statistical Analysis

Statistical analyses were performed using GraphPad Prism 7.0 (GraphPad, USA). Results were expressed as mean ± SD. All data were analyzed by ANOVA followed by Newman–Keuls posttest or a Student t test, according to the characteristics of each experiment. The *p* values <0.05 were considered statistically significant.

## Results

### The Infection of BALB/c Mice With *L. braziliensis* Has Natural Resolution, Whereas Animals Infected With *L. amazonensis* Develop Progressive Lesions

**Figure 1:**
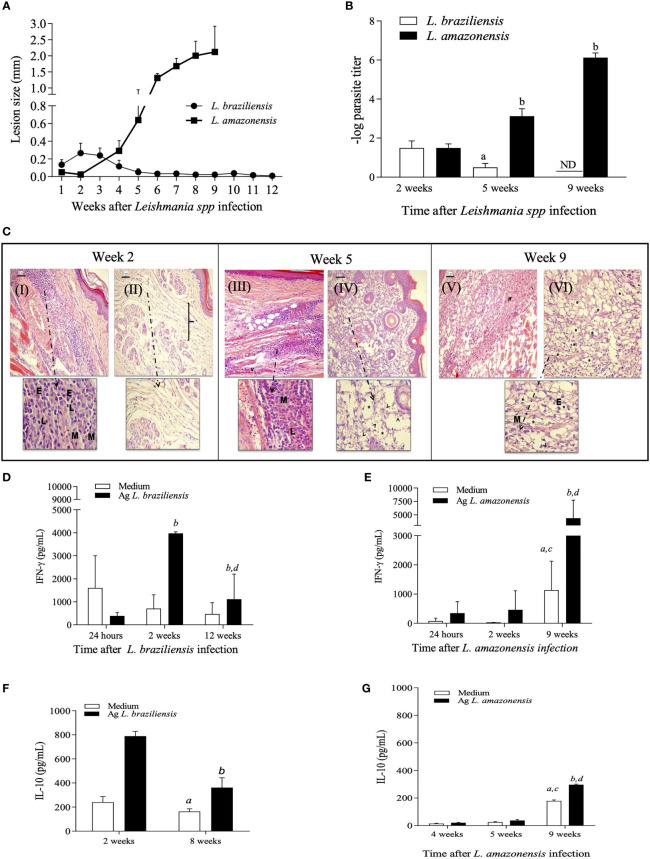
Kinetic evaluation of lesion size, tissue parasitism and histopathological changes during *L. amazonensis* and *L. braziliensis* infection in BALB/c mice. Females of BALB/c mice were inoculated into the left hind footpad with 1x10^5^ promastigotes of *L. amazonensis* or 1x10^7^ promastigotes of *L. braziliensis*. Data are represented as mean ± SD of at least four mice in each time point. **(A)** The development of the lesion size was monitored weekly, based on the difference in thickness between inoculated and the control paw with a digital caliper. **(B)** Parasite loads were evaluated at different times after infection. Statistically significant differences were assumed when p < 0.05 in relation to 2, 5 and 9 weeks after *L. braziliensis* infection and 2, 5 and 9 weeks after *L. amazonensis* infection. **(C)** Histopathological analyses (H&E) were performed 2-, 5- and 9-weeks post-infection for *L. braziliensis* (I e III) and *L. amazonensis* (II, IV, V and VI) respectively. E = eosinophils; M = macrophages; L = lymphocytes, bracket: dispersion of collagen fibers. Levels of IFN-g **(D, E)** and IL-10 **(F, G)** in culture supernatants of lymph node cells, at specific time points after *L. braziliensis* or *L. amazonensis* infection, respectively. Ag means *Leishmania* soluble antigen used to stimulate cultures. (a, c) indicate significant differences (p < 0.05) in cytokine levels in cultures with no stimulus (white bars), as compared to 24 hours (a) or 2 weeks (c); (b, d) indicate differences after stimulus (black bars), as compared to 24 hours or 2 weeks, respectively. ND, not detected.

*L. braziliensis* infection in BALB/c mice have been considered a model of cure and resolution of lesions, while infection by *L. amazonensis* is associated with the development of progressive lesions and failure to resolve infection. To validate these profiles, female BALB/c mice were inoculated with 1x10^7^ promastigotes of *L. braziliensis* or 1x10^5^ promastigotes of *L. amazonensis* and the development of lesions was monitored weekly. As expected, mice inoculated with *L. braziliensis* developed discrete lesions that are self-healing while mice infected with *L. amazonensis* were unable to control lesion development (**Figure 1A**). Also, there was an association between lesion size and tissue parasitism at the site of the infection (**Figure 1B**).

To better characterize the local inflammatory response after infection, histopathological analyses of infected paws and cytokine production by draining lymph node cells after *in vitro* restimulation were performed. The data shown in **Figures 1A–G** confirmed that BALB/c mice can activate and control inflammatory responses during *L. braziliensis* infection, with early recruitment of cells to the site of infection and production of IFN-γ, characterizing a self-resolving model of infection. In *L. amazonensis*-infected mice, a delayed onset of the inflammatory responses is observed, with inflammatory infiltrates and high production of IFN-γ only occurring after 9 weeks of infection, leading to overwhelming inflammation with intense parasite burden, which is detrimental to the host.

### Shutdown of ERK1/2 Phosphorylation at Late Stages of *L. amazonensis* Is Associated With Dramatic Effects on the Course of Infection, Parasite Loads and Inflammatory Responses

**Figure 2:**
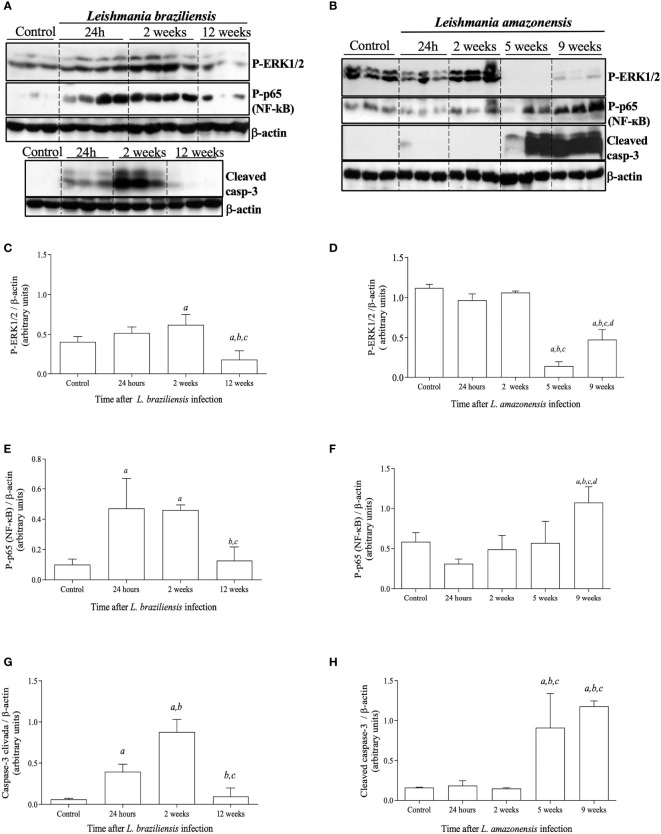
Activation status of signaling proteins involved in inflammation and apoptotic pathways after *L. braziliensis* and *L. amazonensis* infection. Mice were infected in the left hind footpad *with* 1x10^7^ promastigotes of *L. braziliensis* or 1 x 10^5^ promastigotes of *L. amazonensis.* Skin samples from infected footpads were processed and evaluated by Western blotting analysis. **(A)** Western blotting of P-ERK1/2, P-p65/RelA (NF-kB), and cleaved caspase-3 at 24 hours, 2, and 12 weeks after *L. braziliensis* infection. **(B)** Western blotting of P-ERK1/2, P-p65/RelA (NF-kB), and cleaved caspase-3 at 24 hours, 2, 5 and 9 weeks after *L. amazonensis* infection. Densitometric analysis of the correspondent western blots for P-ERK1/2 **(C, D)**, P-p65 (NF-kB) **(E, F)**, and cleaved caspase-3 **(G, H)**, for *L. braziliensis* or *L. amazonensis* infections, respectively. Data are representative of two experiments, with similar results. All data are presented as mean + SD of three mice per group in each time point. Statistically significant differences were assumed when p< 0.05 in relation to control (a), 24 hours post-infection (b), 2 weeks post-infection (c) or 5 weeks post-infection (d).

Aiming to better understand the inflammatory signaling cascades triggered after *Leishmania* infection and how these pathways correlate with the different infection profiles observed between the two species of this parasite, we analyzed the signaling proteins ERK1/2 and NF-κB which are involved on the production of inflammatory mediators and the cleavage of caspase-3 as a marker of apoptosis. In *L. braziliensis-*infected mice, P-ERK1/2 levels are upregulated early at the infection coinciding with the peak of parasitism and inflammation. Accordingly, by the 12^th^ week of infection, when lesions and inflammation were already resolved, P-ERK1/2 levels were found to be decreased (**Figures 2A, C**). No significant alterations in P-ERK1/2 levels were observed either at 24 hours or 2 weeks after infection with *L. amazonensis*, as compared to controls. Interestingly, there was a marked decrease in P-ERK1/2 levels at the fifth week post-infection, which were partially restored at 9 weeks of infection (**Figures 2B, D**). Whether the dramatic decline on P-ERK1/2 levels on the 5^th^ week post *L. amazonensis* infection could be attributed to either degradation or down-regulation of expression of ERK1/2, levels of total ERK1/2 on extracts of *L. amazonensis* infected paws at different time points were assessed. Total ERK1/2 levels in *L. amazonensis* infected mice did not differ from those of control animals, suggesting that there is no degradation or down-regulation of total ERK1/2, during the first five weeks of infection (**Supplementary Figure S1**).

Given that ERK1/2 acts upstream of NF-κB and can activate this transcription factor, which is involved on the production of pro-inflammatory mediators and recruitment and survival of leukocytes (24, 25), we analyzed the *in vivo* activation of NF-κB by phosphorylation of NF-κB-p65*/*Rel*A.* The activation of caspase-3 through the detection of its cleaved products was also analyzed due to its association with cell apoptosis (26, 27). During *L. braziliensis* infection levels of P-p65/Rel*A* and cleaved caspase-3 were increased early at the infection coinciding with the peak of inflammation (**Figures 2A, E and G**). As seen in **Figures 2B, F and H**, levels of P-p65/Rel*A* and cleaved caspase-3 increased significantly after 5 weeks post-*L. amazonensis* infection, alongside with the shutdown of ERK1/2 phosphorylation and at the peak of the infection. These findings suggest that downstream intracellular inflammatory signals that are involved on production of inflammatory mediators or cellular lifespan are activated during *Leishmania* infection and are associated with the temporary shift of P-ERK1/2 responses.

### Inhibition of ERK1/2 Phosphorylation With U0126 *In Vitro* Has Impact on Phagocytosis, Parasite Load and Immune Response

**Figure 3:**
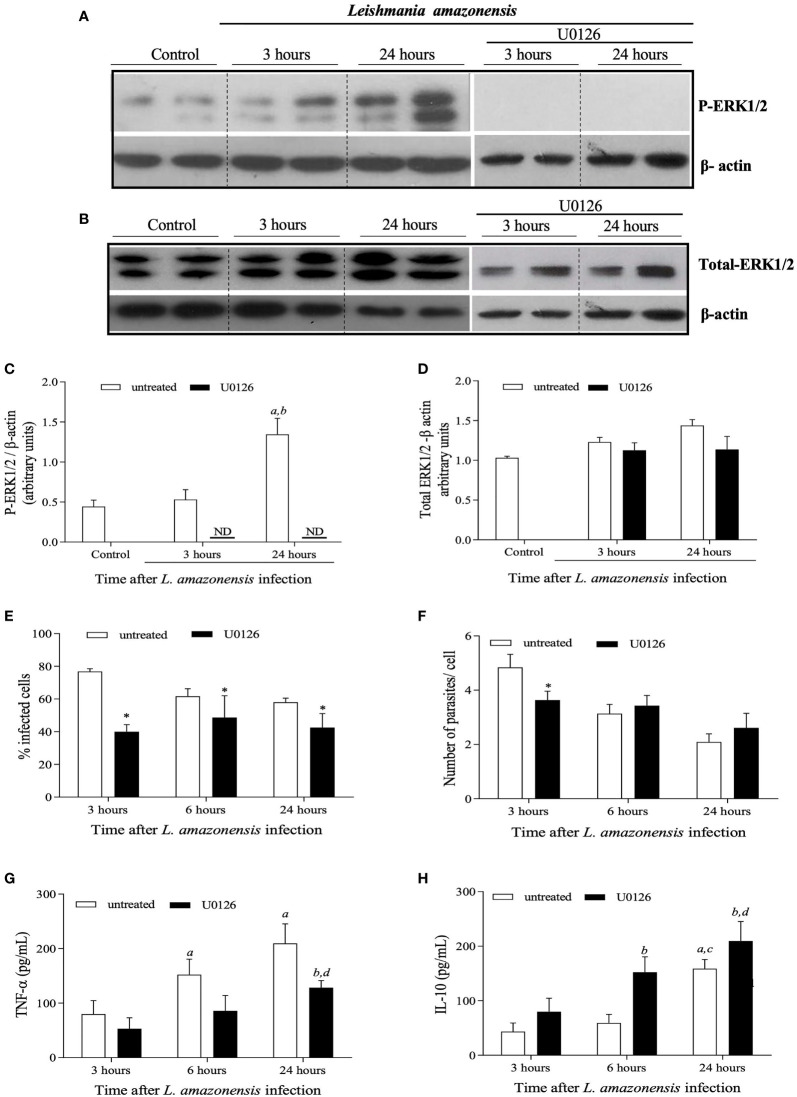
| *In vitro* evaluation of the impact of treatment of BMDMs obtained from BALB/c mice with U0126 on the *L. amazonensis* promastigotes intake and levels of cytokines. BMDMs were pre-treated with 15 mM OF U0126 for 2 hours and then infected with *L. amazonensis* promastigotes, at a 5:1 parasite/cell ratio. Profiles observed in western blotting analysis performed for ERK1/2 phosphorylation **(A)** and total ERK1/2 expression levels **(B)** of not-treated, not infected cells (1) or cells treated with U0126 prior to infection *by L. amazonensis*. Westerns were performed for infected cells either after 3 hours or 24 hours post-infection. Densitometric analyzes for ERK1/2 phosphorylation **(C)** and Total ERK1/2 expression **(D)**, after normalization with β-actin. At 3, 6 and 24h post infection, cells were evaluated for the percentage of infected cells **(E)** and number of parasites per infected cells **(F)**. At the same time points, culture supernatants were assessed for TNF-α **(G)** and IL-10 **(H)** concentrations. Data are representative of two experiments with similar results. In **(C, D)**, statistically significant differences were assumed when p< 0.05 as compared to control cells (a), to 3 hours after infection of untreated cells (b). In **(E, F)**, the asterisk indicates statistically significant differences by comparing infection rates in untreated and treated cells at each time-points after infection. In **(G, H)**, (a) indicates statistically significant differences as compared to control non treated and infected cells for 3 hours and (b) as compared to treated and infected cells for 3 hours and (c) as compared to non-treated and infected cells for 6 hours (d) as compared to treated and infected cells for 6 hours. ND, not detected.

Considering the co-occurrence of P-ERK1/2 shutdown with further increased lesion size, parasite loads and inflammatory responses during *L. amazonensis* infection in BALB/c mice, we hypothesized that the shutdown in ERK1/2 phosphorylation seen around 5 weeks of infection would be a critical event that determines the outcome of infection in this model. To test this hypothesis, we first treated *L. amazonensis*-infected bone marrow-derived macrophages (BMDMs) with U0126, an MEK/ERK1/2 inhibitor. As seen in **Figure 3**, *in vitro* infection of BMDMs with *L. amazonensis* induces increased levels of P-ERK1/2 after 24 hours of culture with no impact on total levels of ERK1/2 (**Figures 3A, B**). Treatment of infected BMDMs with U0126 completely inhibited leishmania induced-ERK1/2 phosphorylation, as shown in **Figures 3A, C**, but did not affect total ERK1/2 levels (**Figure 3D**). The impact of U0126 treatment on the number of *L. amazonensis* infected cells and amastigotes/cell was also evaluated. In the untreated group, the numbers of infected cells, as well as the numbers of amastigotes per cell decrease significantly at 6 and 24 hours, as compared to 3 hours after infection (**Figures 3E, F**, respectively). As shown in **Figure 3E**, treatment with U0126 significantly affected the number of infected cells, shortly after infection (3 hours), which was lower in this group at this time point, as compared to untreated cells. After that, the numbers of infected cells remained constant in the treated group in all time points. Similarly, the numbers of amastigotes per cell remained constant throughout the time points analyzed (**Figure 3F**) in this group, and no significant differences were detected by comparing the values at 3, 6 or 24 hours after infection. These data suggest that the lack of ERK1/2 phosphorylation affects macrophage phagocytosis competence, but not the replication of *L. amazonensis* amastigotes. We next evaluated the effects of U0126 treatment on expression of TNF-α and IL-10 (**Figures 3G, H**). Although TNF-α levels increased in both treated and not treated groups, cells pretreated with U0126 produced significantly lower levels of this cytokine at all time points post-*L. amazonensis* infection, as compared to control cells (**Figure 3G**). IL-10 levels also increased in both groups, as compared to levels detected at 3 hours post infection, but in contrast increased levels were observed in the treated group as compared to levels produced by untreated cells (**Figure 3H**).

Treatment of BMDMs with selumetinib, a highly selective MEK/ERK inhibitor, was used to test specificity and to validate the results obtained with U0126. There was a reduction in the percentage of treated infected cells when compared to untreated cells. The number of parasites per cell was lower at 3 hours in the selumetinib group, but no differences were observed for 6 or 24 hours (**Supplementary Figure S2**). Taken together our *in vitro* data have shown ERK1/2 activation plays a role in macrophage phagocytosis during *L. amazonensis* infection.

### *In Vivo* Inhibition of MEK/ERK During *L. amazonensis* Infection Results in Increased Lesion Size and Parasite Burden

Based on the hypothesis that the shutdown of ERK1/2 phosphorylation around the 5^th^ week of infection is critical for further alterations on the course of *L. amazonensis* infection, and progressive disease, we tested whether administration of U0126, earlier than at the 5^th^ week of infection, would anticipate the onset of lesion development and the increase parasite loads and inflammatory responses in *L. amazonensis* infected mice.

**Figure 4:**
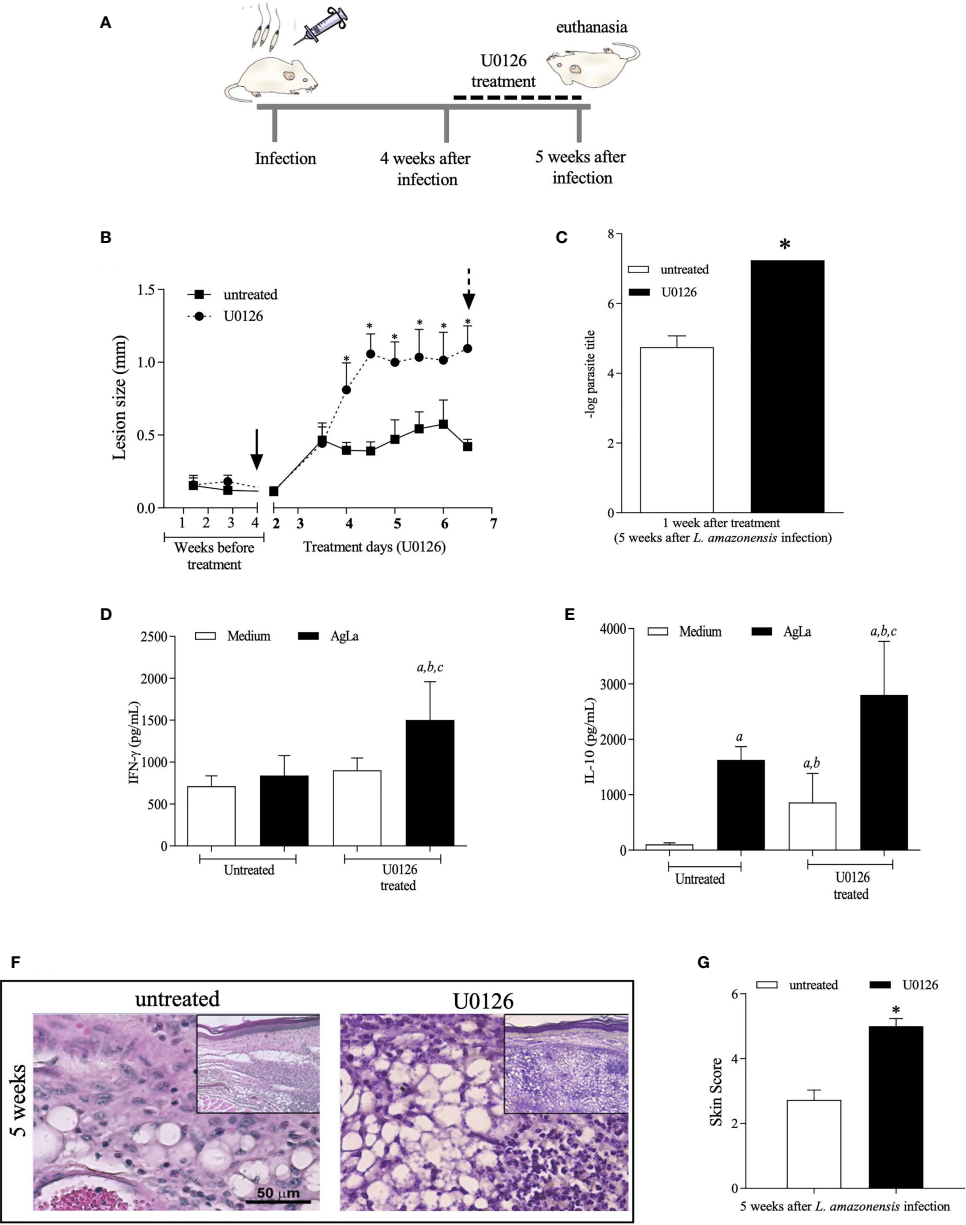
Evaluation of treatment with U0126 at 4 weeks of *L. amazonensis* infection. **(A)** Females of BALB/c mice (n = 6) were inoculated into the left hind footpad with 1x10^5^ promastigotes of *L. amazonensis* and after four weeks of infection animals received U0126 (3mg/kg) intraperitoneally for seven consecutive days and then they were euthanized (5th week). **(B)** The development of the lesion size was monitored weekly, before treatment beginning, based on the difference in thickness between inoculated and the control paw with a digital caliper, and daily, after treatment beginning for seven consecutive days. Black arrow indicates treatment beginning and dotted arrow indicates end of treatment. **(C)** Parasite loads between treated and not treated groups at five weeks after infection. Levels of IFN-g **(D)** and IL-10 **(E)** in culture supernatants of lymph node cells, at end of treatment (5 weeks). Ag means *Leishmania* soluble antigen used to stimulate cultures. Significant differences (p > 0.05) are indicated by (a) as compared to untread and not stimulated cultures, (b) to untread and stimulated cultures and (c) treated and stimulated cultures. **(F)** Histopathological analyses (H&E) were performed at 5 weeks post *L. amazonensis* infection in not treated and untreated groups. Quantification of inflammatory infiltrate (skin scores) in mice footpad at 5 weeks post-infection **(G)**. The asterisks indicate statistically significant differences between treated and untread groups (p < 0.05).

In the first round of experiments, mice were infected and treated for one week, beginning at the fourth week of infection (see experimental design in **Figure 4A**). Administration of U0126 inhibited ERK1/2 phosphorylation while total ERK1/2 levels were not affected during infection with *L. amazonensis* (**Supplementary Figure S3**). These mice were also monitored for lesion development and parasite loads and, as expected, lesion size increased faster in treated mice, as compared to controls (**Figure 4B**). Tissue parasitism was also significantly higher in treated mice at 5^th^ week post infection, as compared to untreated mice (**Figure 4C**).

We also evaluated the production of specific IFN-γ and IL-10 by lymph node cells of infected mice. Interestingly, levels of IFN-γ were higher on cells of treated-infected mice stimulated with the antigenic extract of *L. amazonensis* promastigotes (**Figure 4D**), as compared to untreated animals. In the same line, the specific IL-10 production from cells of treated mice was significantly higher when compared to levels produced by non-stimulated cells or by stimulated cells of untreated mice (**Figure 4E**) suggesting that inhibition of MEK/ERK impacts the inflammatory response influencing cytokine release by lymph node cells of infected mice.

Since our previous data revealed that expressive histopathological changes are observed concurrently with the decreased levels of P-ERK1/2 at the 5^th^ week post-*L. amazonensis* infection, a histopathology analysis was performed in skin tissue sections of U0126 treated and untreated mice. Whilst the skin sections of untreated mice displayed histopathological aspects comparable with that previously reported for 5^th^ week infected mice, skin sections of U0126 treated mice showed worsening of inflammatory responses, which were comparable to those observed after nine weeks of infection in untreated mice (**Figure 4F**). The histological aspects of the lesions were translated in scores, which were significantly higher in treated mice (**Figure 4G**).

**Figure 5:**
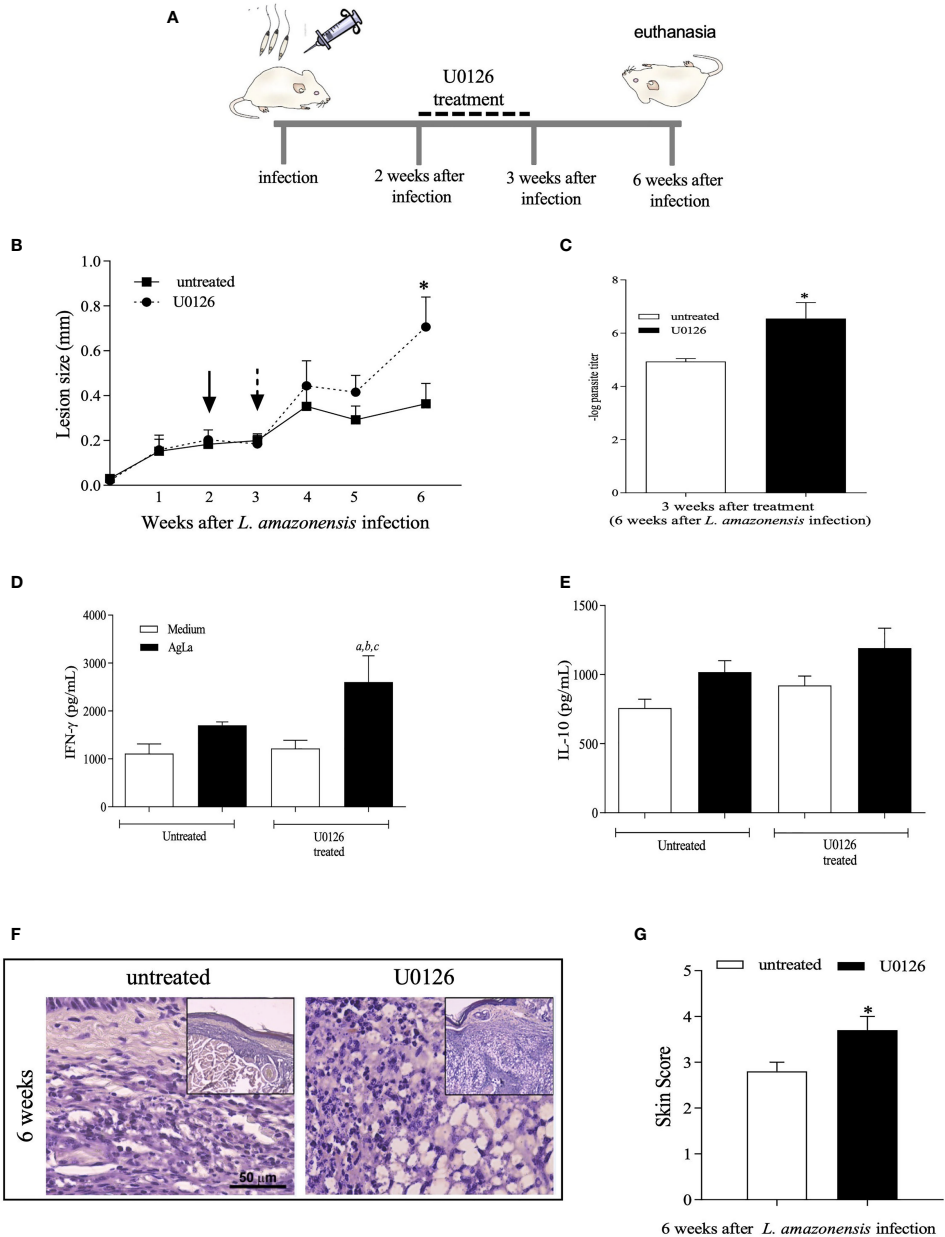
Evaluation of treatment with U0126 at 2 weeks of *L. amazonensis* infection. **(A)** Females of BALB/c mice (n = 6) were inoculated into the left hind footpad with 1x10^5^ promastigotes of *L. amazonensis* and after two weeks of infection animals received U0126 (3mg/kg) intraperitoneally for seven consecutive days. **(B)** The development of the lesion size was monitored weekly, before and after treatment, for 6 weeks, based on the difference in thickness between inoculated and the control paw with a digital caliper. Black arrow indicates treatment beginning and dotted arrow indicates end of treatment. **(C)** Parasite loads between treated and not treated groups, three weeks after end of treatment (six weeks post-infection). Levels of IFN-g **(D)** and IL-10 **(E)** in culture supernatants of lymph node cells 6 weeks after infection. Ag means *Leishmania* soluble antigen used to stimulate cultures. Significant differences (p < 0.05) are indicated by (a) as compared to untread and not stimulated cultures, (b) to untread and stimulated cultures and (c) treated and stimulated cultures. **(F)** Histopathological analyses (H&E) were performed at 6 weeks post *L. amazonensis* infection in not treated and untreated groups. Quantification of inflammatory infiltrate (skin scores) in mice footpad at 6 weeks post-infection **(G)**. The asterisks indicate statistically significant differences between treated and untread groups (p < 0.05).

We also applied a different protocol by administering mice with U0126 earlier after *L. amazonensis* infection, from the 2^nd^ to 3^rd^ week post-infection, and followed up until the 6^th^ week of infection (**Figure 5A**). Inhibition of ERK1/2 phosphorylation by U0126 was observed at the endpoint of the experiment without changing the total levels of ERK1/2 (**Supplementary Figure S4**). Nonetheless, similar effects as those described for treatment at 4^th^ week were observed. Significant increases in lesion size (**Figure 5B**), parasite loads (**Figure 5C**), specific IFN-γ levels (**Figure 5D**) and skin scores of inflammatory responses (**Figures 5F, G**) were detected on U0126-treated mice as compared to controls. Specific IL-10 levels were not significantly altered (**Figure 5E**). Altogether, our data show that short-term inhibition of ERK1/2 phosphorylation seems to be a critical event that modulates innate and adaptive inflammatory responses, during *L. amazonensis* infection, impacting the infection outcome, parasite loads and uncontrolled infection.

## Discussion

*L. amazonensis* and *L. braziliensis* are listed among the most prevalent species causing different clinical manifestations of tegumentary leishmaniasis and have been associated to quite distinct immunopathology profiles, including significant differences in activation of inflammatory responses (2). Although previous studies have investigated the association of the ERK1/2 signaling pathway and inflammatory responses during *Leishmania* spp. infections, our study is the first to comparatively investigate the kinetics of these aspects and their association with the outcomes of *L. braziliensis* and *L. amazonensis* infections in the same host.

Previous data from our group have shown that *L. braziliensis* infection can induce ERK1/2 activation at the site of infection in BALB/c mice (22). ERK activation has also been shown in peritoneal macrophages from BALB/c mice shortly after stimulation with *L. braziliensis* lipophosphoglycan (LPG) (28). Here we showed that ERK1/2 phosphorylation not only is induced in *L. braziliensis* infection, but it correlates with the peak of infection, when we see lesion development, parasite presence and high production of cytokines. For *L. braziliensis* we could argue that ERK signaling is important for generation of protective immune responses that promotes parasite clearance as well as regulation of excessive inflammation. In contrast, we observed a very unusual and distinct profile for ERK1/2 phosphorylation during our *in vivo* studies of *L. amazonensis* infection. While at early stages of infection, only basal levels of P-ERK are detected, a brief shutdown in ERK1/2 activation at around five weeks after infection stands out as crucial event, since parameters associated with disease outcome such as edema, inflammatory infiltrate, and tissue parasitism at the site of infection were altered only after this signaling. Moreover, levels of total ERK1/2 proteins were not altered, indicating that the kinase activity involved in this pathway may be the target for this phenomenon.

Caspase-3 and NF-κB play important roles in the development of inflammatory responses and both these molecules can be activated by several mediators, thus potentiating their effect on immune responses (25, 27). Therefore, it was to be expected that activation of these pathways follows the course of inflammation during *Leishmania* infection. For *L. braziliensis*, activation of caspase-3 and NF-κB can be seen as early as 24 hours after infection, reaching highest levels at 2 weeks, concomitantly with the peak of parasite load and inflammation. For *L. amazonensis*, the peak of parasite proliferation and inflammatory responses occurs at 9 weeks after infection, when, accordingly, the highest levels of cleaved caspase-3 and NF-κB are observed.

The inhibition of host ERK1/2 activation is consistent with the hypothesis that *L. amazonensis* uses non-activation of inflammatory responses to evade host defenses, allowing the entry and free multiplication of the parasite at early stages of infection. Together with the baseline levels of ERK1/2, this hypothesis is corroborated by the absence of NF-κB, caspase 3 and inflammatory infiltrates, at the early stages on the site of the infection. However, the pronounced reduction in P-ERK1/2 levels in the fifth week after infection may be a trigger for this process to be reversed, since only after that macroscopic lesions, inflammatory cell infiltrates and increases in NF-κB and cleaved caspase-3 levels could be observed. Since at this point, parasites loads were already high, it is possible that this process only initiates when the parasitic burden reaches a certain limit, generating signals that would inhibit ERK1/2 phosphorylation, and consequently the modulation of the other various components downstream ERK activation that participate in the signaling cascade. It is still uncertain, however, which signal or what would be the origin of mechanism that mediates dephosphorylation of P-ERK1/2.

How parasites avoid early host cell activation is an intriguing aspect of the course of *L. amazonensis* infection and could be attributed to the capacity of *Leishmania* to modulate host cell signaling pathways, through its virulence factors, as a strategy for survival (29). Consistent with this view, it has been previously demonstrated that LPS-primed peritoneal macrophages inhibited ERK1/2 phosphorylation immediately after infection with amastigotes of *L. amazonensis*, a status that could be reverted by adding a tyrosine phosphatase inhibitor prior to infection (30). In addition, *L. amazonensis* amastigotes can suppress activation of STAT2 (the signal transducer and activator of transcription 2) and induce protein degradation of STAT2 *in vitro*, most likely, through the action of proteases, since parasite lysates alter STAT2 phosphorylation in BMDCs and pretreatment with protease inhibitors is able to block this effect (31).

The silent phase of the *L. amazonensis* infection has also been associated with the presence of T regulatory (Treg) cells at earlier time-points. These cells accumulate during the first two weeks of infection and most likely help to set a permissive environment for parasite replication. As parasite loads increase along the infection, numbers of Treg cells gradually drop (32). To our knowledge, there is no study in the literature that looked into a possible effect of inhibition of ERK activation on Treg cells during *Leishmania* infection. A few *in vitro* studies in different settings have reported upregulation of Foxp3 expression in naïve and activated CD4^+^ T cells after treatment with U0126 (33, 34). How inhibition of ERK activation would affect Treg cell function in *Leishmania* infection is still not understood and should be further explored in future studies.

Modulation of MAPK signaling has been also shown for other pathogens, through different mechanisms. A time-dependent inhibition of ERK1/2 in macrophages infected with a virulent strain of *Yersinia enterocolitica* has been demonstrated, which was also accompanied by lower production of TNF-**α** (35). In human lepromatous leprosy, a form of Hansen’s disease characterized by unresponsiveness of host T cells to the causative agent *Mycobaterium leprae*, infection promotes basal low levels of kinase activity and consequently lower ERK phosphorylation. This modulation in ERK1/2 has been linked to the blockage in IL-2 production (a feature of anergic cells) and the impaired activation of T cells (36). *Shigella flexneri* also displays a well-described mechanism of MAPK modulation that involves gene-specific epigenetic modifications and phosphatase activity of a virulence factor (37, 38). *Leishmania* can also activate the p38 MAPK cascades in *in vitro* infections of macrophages, as reported by a few studies (39–41). Interestingly, activation of p-38 following *Leishmania* infection seems to accompany ERK activation in similar fashion, even if their roles in the induction of immune functions in macrophages are shown to be reciprocal (39, 41). Taking in consideration that the MAPK pathway encompasses a multitude of both upstream activators and downstream targets of ERK1/2 (42), the mechanism suppressing phosphorylation and its precise spatiotemporal dynamics is still uncertain in our model. Nonetheless, these factors seem to be crucial for regulating and maintaining breadth and extent of signaling during *L. amazonensis* in BALB/c mice.

Among the possible mechanisms of regulation of ERK1/2 during infection, we may highlight the secreted phosphatase activity of *Leishmania* parasites. Particularly, *L. amazonensis* amastigotes have been shown to secrete both acid and basic phosphatases after infection of macrophage cultures (35). It is also known that several *Leishmania* species are able to activate important negative regulators of MAPK signaling pathway, the protein tyrosine phosphatases (PTPs), such as SHP-1 and PTP-1B, leading to attenuation of innate immune responses and the consequent reduced production of leishmanicidal molecules (43–46). Moreover, the *Leishmania* surface protease gp63, which is internalized by macrophages soon after the first contact with the parasites, can alter the profile and activate SHP-1, PTPB1 and other host proteases by proteolytic cleavage. Interestingly, BALB/c mice deficient in PTPB1 infected with *L. major* showed a delay in lesion development, during the first five weeks of infection, suggesting a role for this host protease in disease progression at early infection time-points (45). Lag in lesion development can also be observed in BALB/c mice infected with a *L. amazonensis* mutant in which gp63 expression is downregulated (47). Thus, it is plausible that *L. amazonensis* utilize virulence factors such as secreted phosphatases or internalized proteases to modulate the MAPK pathway and evade the immune system at early stages of infection.

While the natural trigger responsible for the shutdown of ERK1/2 phosphorylation during *in vivo L. amazonensis* infection is still uncertain, we used the compound U0126, which selectively inhibits phosphorylation of ERK1/2 (48), to treat *L. amazonensis* infected mice. We hypothesized that if performed before the natural decrease in P-ERK1/2 levels, this intervention would anticipate the effects of the shutdown on ERK1/2 phosphorylation, and consequently would alter the course of inflammatory responses and the infection outcome. As expected, the administration of U0126 inhibited ERK1/2 phosphorylation, anticipated the progression of *L. amazonensis* infection in BALB/c mice, and reproduced the alterations seen naturally in untreated mice. This impact was observed even when U0126 was administered as early as 2 weeks post-infection. In parallel, inhibition of ERK1/2 activation in BMDMs infected with *L. amazonensis* by using U0126, reduced phagocytosis and the levels of TNF-α, but increased IL-10 levels, in treated cells.

Conversely to our findings, a previous study has observed smaller lesions and decreased parasite burden after administration of U0126 to BALB/c mice infected with *L. amazonensis* (17). These distinct profiles could be explained, at least in part, by differences in the protocols that were applied, regarding the duration of stimulation, time after infection that the administration was initiated and the dose of the inhibitor. However, a marked difference between the protocols seems to be the use in our study of axenic promastigotes for infection and not tissue amastigotes, as reported by Yang et al. (17). Amastigotes derived from lesions have IgG on their surface making more likely that they are opsonized and internalized through Fc receptor, which may promote a completely distinct profile for activation of inflammation, including the rapid activation of ERK and immune responses. It has been shown that Fc-mediated activation of NF-κB, which regulates the transcription of many genes expressed during inflammatory responses, following phagocytosis of microbes, requires MAPK signaling (17, 49, 50). Axenically grown and not opsonized amastigotes failed to activate ERK and to induce IL-10 production, but opsonization of amastigotes with IgG restored their ability to activate ERK and induce IL-10 (17), which can be regulated by ERK1/2 phosphorylation. Production of this anti-inflammatory mediator during *Leishmania* infection can affect production of microbicidal molecules such as nitric oxide (51–53). Indeed, Yang et al. (17) reported high levels of P-ERK1/2 at early stages of infection (3 weeks) while, at the same time-point, only basal levels of P-ERK1/2 could be observed in our model. In addition, Yang et al. used U0126 to inhibit ERK1/2 activation when IL-10 levels were already high, while in our experiments administration of U0126 was performed prior to the increase in parasite specific IL-10 levels or other adaptive immune responses, thus mimicking the natural phenomenon of temporary shutdown on ERK activation.

Inhibition of ERK1/2 activation stimulates the production of IL-12 (54). Since IL-12 is critical for antigen presentation and induction of adaptive immune responses during *Leishmania* infection, including the production of IL-2 by CD4^+^ T cells, it is plausible to hypothesize that a late and temporally induction of IL-12, through the brief shutdown in ERK1/2 activation, could be an alternative mechanism to switch the patterns of inflammatory and adaptive immune responses at late stages of *L. amazonensis* infection. Additional experiments are, however, necessary to further clarify this hypothesis.

MAPK inhibitors have been proposed as potential treatments for inflammatory disorders such as asthma, rheumatoid arthritis, and cancer (55–57). However, our data suggest that, for *L. amazonensis* infection, that would not be the case. In contrast, phosphatase inhibitors may be an interesting alternative, since phosphatases are important virulence factors for *Leishmania* infections (43, 44). A few studies have looked at the role of phosphatase inhibitors in *Leishmania* infection as means to modulate immune responses through ERK phosphorylation (58, 59). Inhibition of phosphatase activity, at convenient time-points during *L. amazonensis* infection, could prove useful as a potential target for the development of therapeutic strategies. Nonetheless, these alternatives are far from being fully examined to treat *Leishmania* infection, and further studies are needed to better understand the mechanisms that may promote adequate activation of inflammatory and adaptive responses and how they may be explored for therapeutic purposes.

DCL patients infected by *L. amazonensis* show impairment of Th1 immune responses with decreased production of IFN-γ, are refractory to several drug therapies, display uncontrolled parasitism in lesions, highlighting a relative state of immunosuppression (60, 61). Currently there is no vaccine approved for human use against tegumentary leishmaniasis, even though many different preparations have been studied for the last few decades. The anergic nature of the response in DCL may be of relevance for the development of effective vaccine strategies against *L. amazonensis* infection, although a complete picture of host-pathogen interactions is not fully understood for this disease (62). T cell suppression during *L. amazonensis* infection has been associated to impairment of the ability of infected antigen presenting cells (APCs) to process and present parasite antigens (63, 64). Given that MAPK signaling has been shown to be involved in various immune functions including maturation of APCs (65) and antigen presentation (66), successful induction of protection by any vaccination strategy against *L. amazonensis* may therefore be dependent upon modulation of ERK1/2 activation.

In summary our findings bring to light a previously not reported event in *L. amazonensis* infection in BALB/c mice related to a temporary shutdown of ERK1/2 phosphorylation, driven the susceptibility phenotype to infection by this parasite. The results presented here provide a further understanding of the modulation of the ERK1/2 pathway during *L. amazonensis* infection as a mechanism to allow initial parasite multiplication, delay on inflammatory and adaptive T cell responses, to guarantee parasite survival and progressive infection. Further exploring of these associations could be important to optimize the development of successful vaccines and therapeutic interventions against *L. amazonensis* infection.

## Data Availability Statement

The raw data supporting the conclusions of this article will be made available by the authors, without undue reservation.

## Ethics Statement

The animal study was reviewed and approved by Comissão de Ética no uso de Animais (CEUA) of the Federal University of Minas Gerais under the protocol number 240/2016.

## Author Contributions

AF, LPS, and MS-T designed research, analyzed data, and wrote the manuscript. LO, TR, FC, KL, JV, and MS-T performed experiments. CQ-J and LO performed the histopathological analysis. LS critical analyzed the data and wrote the manuscript. All authors contributed to the article and approved the submitted version.

## Funding

This work was supported by the Instituto Nacional de Ciência e Tecnologia em Vacinas (INCTV)/CNPq (grant nº 465293/2014-0); Rede Mineira de Biomoléculas (FAPEMIG grant nº red001214). Authors received research and students fellowships from Conselho Nacional de Desenvolvimento Científico e Tecnológico (CNPq, Brazil) and Coordenação de Aperfeiçoamento de Pessoal de Nível Superior (CAPES, Brazil), respectively.

## Conflict of Interest

The authors declare that the research was conducted in the absence of any commercial or financial relationships that could be construed as a potential conflict of interest.

## Publisher’s Note

All claims expressed in this article are solely those of the authors and do not necessarily represent those of their affiliated organizations, or those of the publisher, the editors and the reviewers. Any product that may be evaluated in this article, or claim that may be made by its manufacturer, is not guaranteed or endorsed by the publisher.

## Supplementary Material

Supplementary Figure S1 Levels of total ERK 1/2 expression in mice footpads after *L. amazonensis* infection. Mice were infected in the left hind footpad with 1 x 10^5^ promastigotes of *L. amazonensis*. **(A)** At 24 hours, 2-, 5- and 9-weeks post infection skin samples from infected footpads were processed and evaluated by Western blotting for analysis of total ERK1/2. For loading control, membranes were reprobed with β-actin. **(B)** Densitometric analysis of the correspondent western blots for total-ERK1/2 at different time-points after *L. amazonensis* infections. Significant differences in total ERK1/2 levels (p < 0.05) were observed at 9 weeks post infection, as compared to control non-infected (a), 2 weeks (b), 5 weeks (c) and 6 weeks post-infection (d).

Supplementary Figure S2 -*In vitro* evaluation of the impact of treatment with selumetinib on parasite intake in BMDMs from BALB/c mice, after *L. amazonensis* infection. BMDMs were pre-treated with 15 mM of selumetinib for 2 hours and then BMDMs from BALB/c mice were infected with *L. amazonensis* promastigotes at a 5:1 parasite/cell ratio. At 3-, 6- and 24 -hours post-infection cells were evaluated for percentage of infected cells **(A)** and number of parasites per infected cells **(B)**. Statistically significant differences (p< 0.05) are indicated by an asterisk.

Supplementary Figure S3 - Levels of total ERK1/2 and P-ERK1/2 in BALB/c mice footpads after 5 weeks of *L. amazonensis* infection and treatment with U0126. Females of BALB/c mice were inoculated into the left hind footpad with 1x10^5^ promastigotes of *L. amazonensis* and, after four weeks of infection, animals received U0126 (3mg/kg) intraperitoneally for seven consecutive days. Animals were euthanized at 5 weeks post-infection. Western blotting analysis for quantification of P-ERK1/2 **(A)** and total ERK1/2 **(B)** levels in footpads of control uninfected, infected and untreated and U1026 treated mice (3 representative mice per group). Densitometric analyzes of average levels of P-ERK1/2 **(C)** and total ERK1/2, after normalization with β-actin. Statistically significant differences were observed as compared to either control uninfected (a) or infected and untread mice (b).

Supplementary Figure S4 - Levels of total ERK1/2 and P-ERK1/2 in BALB/c mice footpads after 6 weeks of *L. amazonensis* infection and treatment with U0126. Females of BALB/c mice were inoculated into the left hind footpad with 1x10^5^ promastigotes of *L. amazonensis. A*fter two weeks of infection animals received U0126 (3mg/kg) intraperitoneally for seven consecutive days. Animals were euthanized 6 weeks post-infection. Western blotting analysis performed at 6 weeks post-infection for quantification of P-ERK1/2 **(A)** and total ERK1/2 **(B)** levels in footpads of control uninfected, infected and untreated and U1026 treated mice (3 representative mice per group). Densitometric analyzes of average levels of P-ERK1/2 **(C)** and total ERK1/2, after normalization with β-actin. Statistically significant differences were observed as compared to either control uninfected (a) or infected and untread (b) mice.
