# Supplementary figures and images for: Temporary Shutdown of ERK1/2 Phosphorylation Is Associated With Activation of Adaptive Immune Cell Responses and Disease Progression During Leishmania amazonensis Infection in BALB/c Mice
Source: Front Immunol. 2022 Jan 25;13:762080. doi: 10.3389/fimmu.2022.762080 (PMC8821891; doi:10.3389/fimmu.2022.762080)

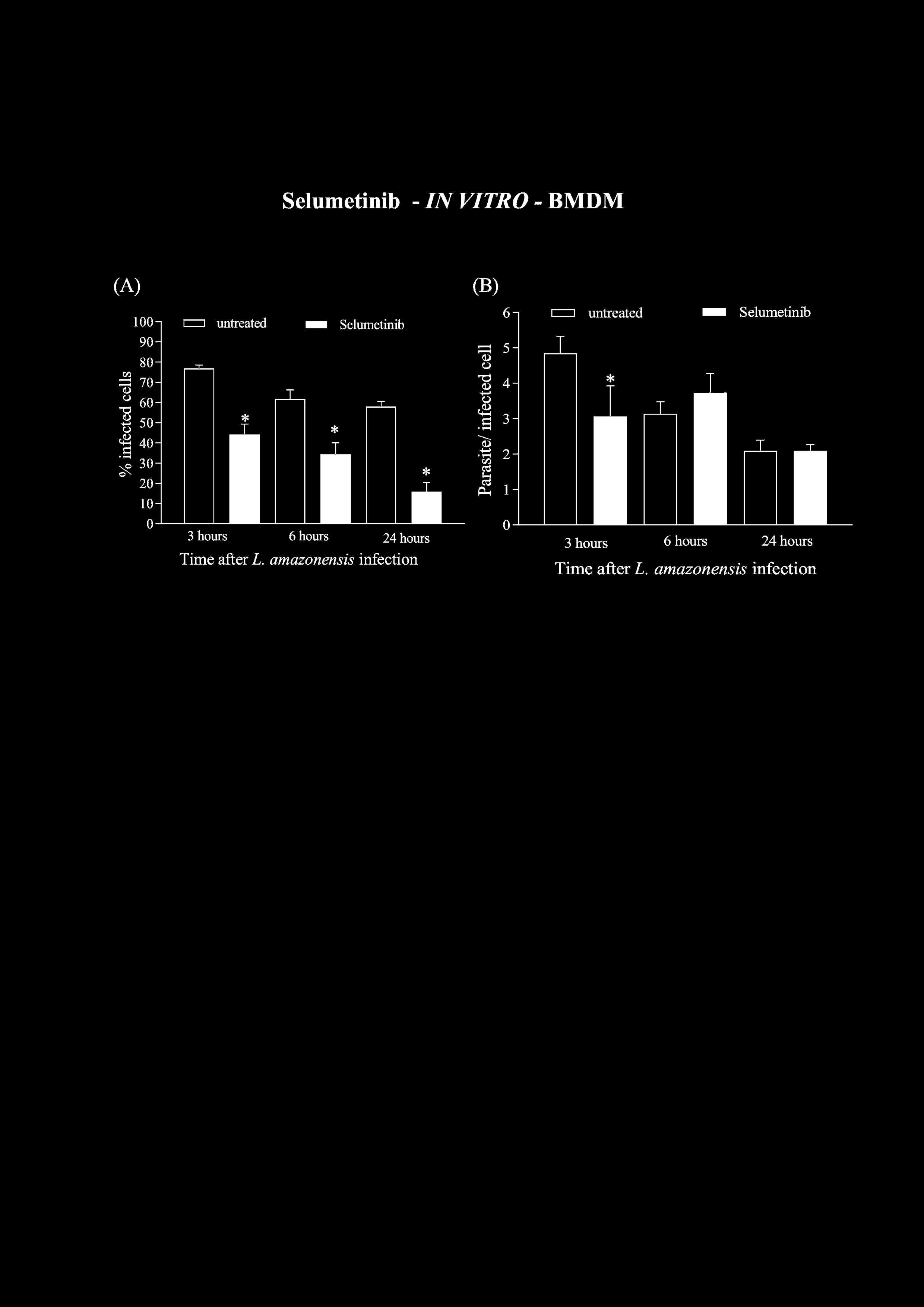

Supplement: Supplementary Figure S2 — -In vitro evaluation of the impact of treatment with selumetinib on parasite intake in BMDMs from BALB/c mice, after L. amazonensis infection. BMDMs were pre-treated with 15 mM of selumetinib for 2 hours and then BMDMs from BALB/c mice were infected with L. amazonensis promastigotes at a 5:1 parasite/cell ratio. At 3-, 6- and 24 -hours post-infection cells were evaluated for percentage of infected cells (A) and number of parasites per infected cells (B). Statistically significant differences (p< 0.05) are indicated by an asterisk. [file Image_2.tiff]
